# Public Awareness and Attitudes Toward Lifestyle Modifications for Controlling Osteoarthritis and Their Relationship to Cardiovascular Risk Factors

**DOI:** 10.7759/cureus.69344

**Published:** 2024-09-13

**Authors:** Reem A AlQarni, Hawra M Aldandan, Zainab Ali Alnahwi, Dhiyaa A Almusaylim, Rana AlQarni, Waad A Alduraywish, Eman Abdullah

**Affiliations:** 1 College of Medicine and Surgery, King Faisal University, Al Ahsa, SAU; 2 Rheumatology, King Faisal University, Al Ahsa, SAU

**Keywords:** cardiovascular risk factors, chronic disease management, health education, lifestyle modification, osteoarthritis, patient attitudes, preventive healthcare, public awareness, rheumatology

## Abstract

Background

Osteoarthritis (OA), common in older adults, leads to joint degradation and inflammation, with risk factors including age, obesity, and genetics. OA strongly indicates cardiovascular disease (CVD), with shared risk factors such as lack of exercise and muscle weakness. This study explores the awareness of OA and its relationship to CVD risk factors.

Methodology

This cross-sectional study in Al Ahsa, Saudi Arabia, involved an online self-administered questionnaire distributed via Google Forms to randomly selected adults, with ages ranging from 18 to 65 years. Informed consent was taken from all participants. Data was cleaned in Excel and analyzed using SPSS version 29 (IBM Corp., Armonk, NY, USA).

Results

Our study assessed the awareness of 381 participants about the relationship between OA and CVD. Most participants were female 312 (81.9%). Overall, 250 (65.6%) participants were aged 18-27, and 362 (95.0%) were Saudi nationals. Further, 210 (55.1%) participants had a bachelor’s degree, and 223 (58.5%) were students. Healthcare exposure varied, with 207 (54.3%) having no background, and 157 (41.2%) being healthcare students. Notably, 152 (39.9%) knew someone diagnosed with OA, of whom 55 (14.4%) also had CVD. Significant predictors of awareness included studying in healthcare (Exp(B) = 3.325, p = 0.001), receiving OA information (Exp(B) = 2.222, p = 0.007), sources such as school/university (Exp(B) = 7.851, p = 0.000), and personal experience (Exp(B) = 4.768, p = 0.034).

Conclusions

This study showed a notable gap in awareness about the link between OA and CVD in Al Ahsa, Saudi Arabia. While those with healthcare exposure showed good knowledge, many lacked an understanding of their relationship. Targeted education, particularly for younger and non-healthcare-educated groups, is crucial for improving awareness and promoting effective lifestyle modifications to manage both OA and CVD risks.

## Introduction

Osteoarthritis (OA) is a progressive joint disorder characterized by chronic pain with functional disability [1]. Articular cartilage degradation is the hallmark of OA. A breakdown of the cartilage matrix results in the production of fibrils and fissures, the emergence of extensive ulcerations, and the removal of the entire thickness surface of the joint. This is accompanied by thickening of the subchondral plate and abnormalities in bone with the formation of osteophytes. In addition to causing cartilage destruction during the clinical stage of the disease, OA also modifies the synovial membrane, where an inflammatory response is often observed [2]. Age, gender, previous joint injury, obesity, genetic predisposition, and mechanical variables, such as malalignment and aberrant joint shape, are the most frequent risk factors for OA [3,4]. When OA is present in persons 60 years of age and older, it is a strong indicator of cardiovascular disease (CVD) [5]. According to a previous study, compared to 2,905,087 (9%) individuals without OA, 12,266,923 (38%) individuals with the condition have CVD [6]. The onset and progression of symptomatic OA are also linked to traditional risk factors for CVD, such as diabetes, dyslipidemia, hypertension, and obesity. This finding may indicate common pathophysiological processes or pathways in the development of these conditions [7,8].

There are several possible reasons why OA and CVD are related. The first is a lack of exercise. Patients with advanced OA are less physically active than those without arthritis due to excruciating joint pain [9]. According to recent research, arthritis-related immobility may shorten older patients’ lives by raising their risk of CVD [10]. Chronic inflammation is the second reason. Even though OA is frequently described as a degenerative condition, new research indicates that synovial inflammation contributes to the early phases of OA development [11]. Muscle weakness comes in third. Those with OA are more likely than healthy controls to experience muscle weakness [12,13]. It has been noted that among those with CVD, muscle weakness is a comorbid illness and a risk factor [14]. Moreover, non-steroidal anti-inflammatory drugs, which are frequently prescribed to relieve pain related to OA, have been linked to an elevated risk of CVD [15,16].

This study aimed to investigate the degree of awareness and association between lifestyle modifications to control OA and its relationship to cardiac risk factors.

## Materials and methods

Study design

This cross-sectional study was conducted via an online, self-administered questionnaire in Al Ahsa, Saudi Arabia. The questionnaire, developed by the authors, was validated and its reliability was ensured before the study. Informed consent was obtained from all participants before they completed the questionnaire. Details of the questionnaire are provided in the Appendices.

Study population and sampling

The target population was adult residents of Al Ahsa. Participants’ ages ranged from 18 to 65 years. A sample of 381 individuals was selected through a random sampling technique from this population.

Data collection

Data were collected using a structured questionnaire hosted on Google Forms. The validated questionnaire was formulated based on the study objectives. The link was randomly distributed online to the adult population of Al Ahsa.

Ethical considerations

Ethical approval was obtained from the relevant research ethics committee before data collection. Informed consent was obtained from all participants, ensuring confidentiality, anonymity, and voluntary participation. Participants were informed about the purpose of the study. Ethical clearance was granted by the Deanship of Scientific Research at King Faisal University on March 20, 2024 (approval number: KFU-REC-2024-MAR-ETHICS2103).

Statistical analysis

A comprehensive statistical analysis was conducted on the dataset, encompassing both descriptive and inferential methodologies. First, a descriptive analysis was conducted to summarize the demographic characteristics of the participants, which included age, gender, and other features. This provided an overview of the study population. Subsequently, inferential analyses such as the Mann-Whitney U test and the Kruskal-Wallis test were employed to examine the awareness score difference between variables. Binary regression was used to determine the predictor of high awareness. Statistical significance was established at a p-value of 0.05 or lower and a 95% confidence interval. All statistical analyses were performed using SPSS Software, version 29.0.0 (IBM Corp., Armonk, NY, USA).

## Results

The study included 381 participants to assess the awareness of the relationship between OA and CVD. The sample was predominantly female (312, 81.9%) compared to male (69, 18.1%). Most participants were young adults aged 18-27 years (250, 65.6%) and Saudi nationals (362, 95.0%). Regarding education, the largest group held a bachelor’s degree (210, 55.1%), followed by those with secondary education or less (120, 31.5%). Employment status varied, with students comprising the majority at 223 (58.5%), followed by employees at 62 (16.3%). Other groups included housewives (41, 10.8%), retired individuals or others (31, 8.1%), and the unemployed (22, 5.8%). In terms of healthcare exposure, 207 (54.3%) had no healthcare background, while 157 (41.2%) were healthcare students, and 17 (4.5%) were healthcare employees. Notably, 152 (39.9%) reported being diagnosed with or knowing someone diagnosed with OA, and among these, 55 (14.4%) had also been diagnosed with CVD. Additionally, 230 (60.4%) had received information related to joint OA (Table 1).

**Table 1:** Sociodemographic parameters and incidence of OA and CVD among participants. OA: osteoarthritis; CVD: cardiovascular disease

Sociodemographic data		Frequency (n = 381)	Percent
Gender	Female	312	81.9
	Male	69	18.1
Age (years)	18–27	250	65.6
	28–37	44	11.5
	38–47	28	7.3
	48–57	43	11.3
	>58	16	4.2
Nationality	Non-Saudi	19	5.0
	Saudi	362	95.0
Education	Secondary/Less	120	31.5
	Diploma	33	8.7
	Bachelor’s	210	55.1
	Postgraduate	18	4.7
Employment	Unemployed	22	5.8
	Housewife	41	10.8
	Employee	62	16.3
	Student	223	58.5
	Retired/Other	31	8.1
Study/Work in the health field?	No	207	54.3
	Yes (student in healthcare)	157	41.2
	Yes (employee in healthcare)	17	4.5
Diagnosed with/know someone diagnosed with OA	Yes	152	39.9
If yes, ever been diagnosed with CVD	Yes	55	14.4
Ever received information related to joint OA?	Yes	230	60.4

**Figure 1:**
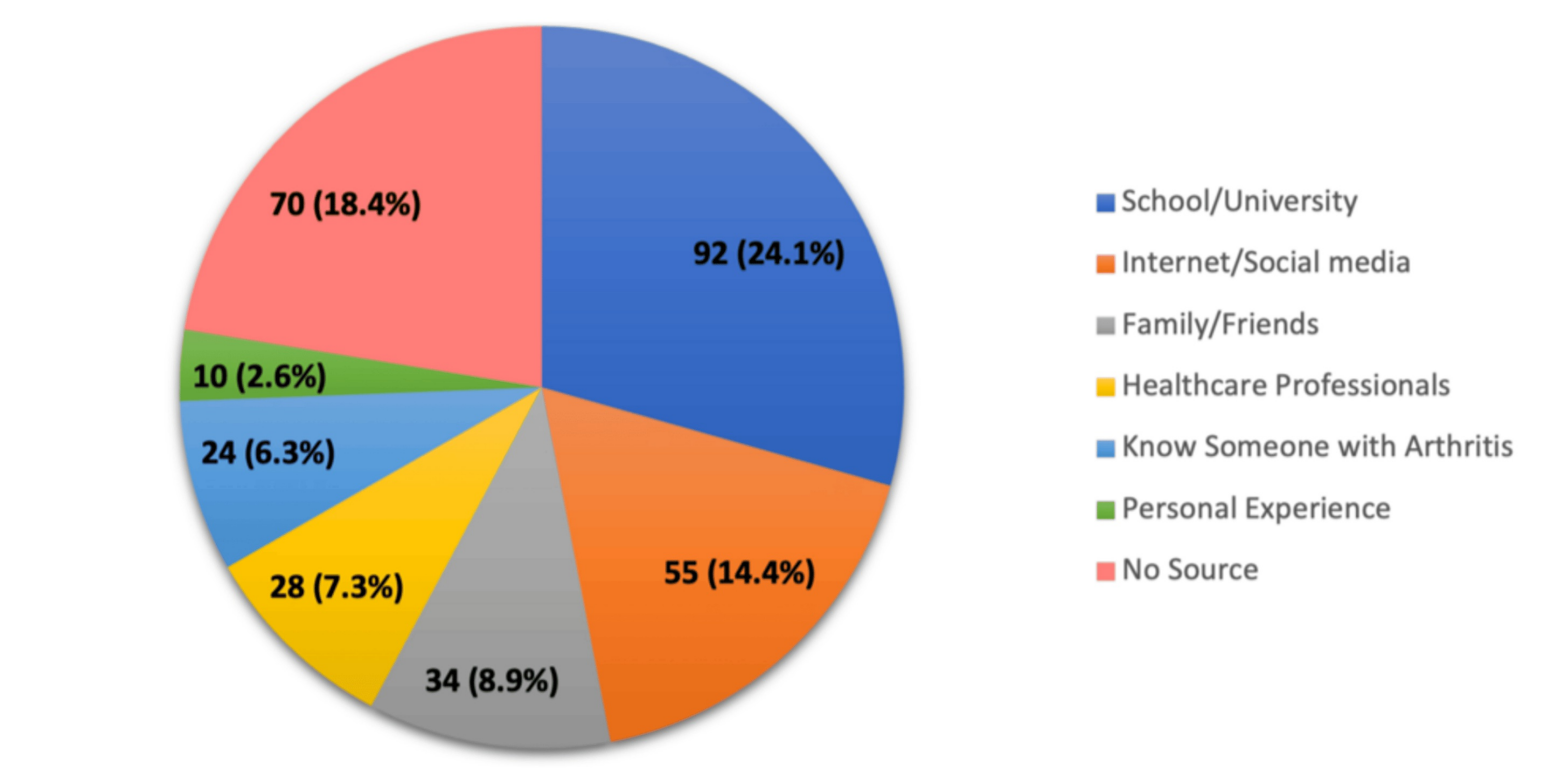
Sources of information about cardiovascular disease and osteoarthritis and their relationship.

Figure 1 shows the sources from which participants received information about CVD and osteoarthritis OA. The most common source was school or university, cited by 92 (24.1%) participants. A significant proportion, 70 (18.4%), reported having no source of information. The internet and social media were the next most frequent sources used by 55 (14.4%) participants. Family and friends were a source for 34 (8.9%) respondents, while healthcare professionals provided information to 28 (7.3%). Additionally, 24 (6.3%) participants gained knowledge from knowing someone with arthritis, and 10 (2.6%) relied on personal experience.

Table 2 shows the results of the multivariate analysis, highlighting significant predictors. Using “No source” as the reference category, participants who cited school or university as their source of information were nearly eight times more likely to have high knowledge (Exp(B) = 7.851, p = 0.000). The internet and social media were also significant sources, increasing the likelihood of high knowledge by over two and a half times (Exp(B) = 2.639, p = 0.009). Knowing someone with OA emerged as a highly significant predictor, making participants over 10 times more likely to have high knowledge (Exp(B) = 10.217, p = 0.000). Personal experience with OA significantly increased the likelihood of high knowledge by nearly five times (Exp(B) = 4.768, p = 0.034).

**Table 2:** Adjusted predictors related to the source of information among participants (multivariate analysis). B: regression coefficient; Sig.: significance; Exp(B): exponentiation of the B coefficient; CI: confidence interval; Ref: reference category; OA: osteoarthritis

Source of information	B	Sig.	Exp(B)	95% CI	
				Lower	Upper
No source	Ref	0.000	Ref	Ref	Ref
School/University	2.061	0.000	7.851	3.862	15.963
Internet/Social media	0.971	0.009	2.639	1.272	5.477
Family/Friends	0.832	0.052	2.299	0.994	5.315
Healthcare professionals	0.858	0.060	2.358	0.964	5.768
Know someone with OA	2.324	0.000	10.217	3.128	33.375
Personal experience	1.562	0.034	4.768	1.128	20.156
Constant	-0.715	0.005	0.489	-	-

Table 3 shows the assessment of knowledge regarding OA causative factors and symptoms. A significant majority (302, 79.3%) were aware that the erosion of cartilage surrounding the joint is a causative factor. Additionally, 189 (49.6%) were aware that decreased circulation around joints is a causative factor. Most respondents (309, 81.1%) understood that decreased synovial fluid with increased movement is related to OA, and 234 (61.4%) acknowledged that increased joint usage among workers is also a factor. Regarding OA symptoms, 70 (18.4%) recognized pain as the only symptom of OA. A majority of 250 (65.6%) identified swelling as a symptom, and 307 (80.6%) acknowledged stiffness as a symptom. Furthermore, 294 (77.2%) understood that OA can lead to a loss of joint movement. Lastly, 206 (54.1%) believed that all joints could suffer from OA.

**Table 3:** Knowledge and causative factors of osteoarthritis among participants.

Knowledge about specific statements		Frequency (n = 381)	Percent
Knowledge about causative factors			
Erosion of cartilage surrounding joint	Don’t know/No	79	20.7
	Yes	302	79.3
Decrease circulation around joints	Don’t know/No	192	50.4
	Yes	189	49.6
Decreases the amount of synovial fluid with increased movement	Don’t know/No	72	18.9
	Yes	309	81.1
Increased joint usage among labors	Don’t know/No	147	38.6
	Yes	234	61.4
Knowledge about signs/symptoms			
Do you think that pain is the only symptom of osteoarthritis?	Don’t know/No	311	81.6
	Yes	70	18.4
Do you think swelling is a sign of joint osteoarthritis?	Don’t know/No	131	34.4
	Yes	250	65.6
Do you think that stiffness is a symptom of osteoarthritis?	Don’t know/No	74	19.4
	Yes	307	80.6
Do you think that joint osteoarthritis can lead to loss of joint movement?	Don’t know/No	87	22.8
	Yes	294	77.2
Do you think that all joints in the body can suffer from osteoarthritis?	Don’t know/No	175	45.9
	Yes	206	54.1

Table 4 shows the lifestyle modifications for controlling OA and their relationship to CVD risk factors. A significant majority (298, 78.2%) recognized the relationship between OA and age. Fewer than half (186, 48.8%) associated OA more with women, and 189 (49.6%) understood that genetics could be a risk factor. Previous joint injuries were identified as a risk factor by 267 (70.1%), while 179 (47.0%) acknowledged joint surgeries as a risk factor. Regarding the connection between OA and CVD, 151 (39.6%) recognized a relationship in terms of risk factors. Further, 277 (72.7%) believed that muscle weakness and joint stiffness could lead to inactivity and, consequently, to CVD. Obesity was seen as a contributing factor to both OA and CVD by 313 (82.2%). Additionally, 256 (67.2%) thought that an unhealthy diet and inadequate water intake could cause joint stiffness and lead to CVD. High cholesterol levels were acknowledged by 230 (60.4%) participants as a cause of both conditions, while 209 (54.9%) and 210 (55.1%) recognized hyperglycemia and smoking, respectively, as shared risk factors. Diagnostic methods for OA, such as clinical examination and X-rays, were known to 235 (61.7%), but only 114 (29.9%) believed OA could be diagnosed using laboratory tests. Regarding treatment, 261 (68.5%) emphasized the importance of weight reduction, a healthy diet, and adequate hydration. Controlling risk factors to reduce CVD incidence was recognized by 247 (64.8%) participants. Painkillers were considered a treatment by 124 (32.5%), while physical therapy and regular exercise were seen as effective by 300 (78.7%). Interventional treatments with cortisone and hyaluronic acid and stem cells were acknowledged by 173 (45.4%) and 143 (37.5%), respectively, and joint replacement surgery for advanced cases was recognized by 266 (69.8%) participants.

**Table 4:** Assessment of awareness and association between lifestyle modifications to control OA and their relation to cardiovascular risk factors. OA: osteoarthritis; CVD: cardiovascular disease; HA: hyaluronic acid

Variables		Frequency (n = 381)	Percent
OA is related to age	Yes	298	78.2
OA is associated more with women?	Yes	186	48.8
Genetics can be a risk factor for developing OA	Yes	189	49.6
Previous joint injuries are a risk factor for developing OA	Yes	267	70.1
Joint surgeries are a risk factor for developing OA	Yes	179	47.0
Any relationship in terms of risk factors between CVD and OA	Yes	151	39.6
Muscle weakness/Joint stiffness is a reason for a lack of activity and leads to CVD	Yes	277	72.7
Obesity can lead to OA of the joints and thus lead to CVD	Yes	313	82.2
Unhealthy diet/Not drinking water causes joint stiffness and leads to CVD	Yes	256	67.2
High levels of cholesterol in the blood can be a cause of CVD and OA	Yes	230	60.4
Hyperglycemia could be a risk factor for CVD and OA	Yes	209	54.9
Smoking/Sitting with smokers causes CVD and joint OA	Yes	210	55.1
OA is diagnosed through clinical examination and regular diagnostic X-rays	Yes	235	61.7
Do you think that OA can be diagnosed using laboratory tests?	Yes	114	29.9
Treating OA is achieved by reducing weight, healthy diet, and drinking water	Yes	261	68.5
Controlling risk factors that cause joint OA reduces the incidence of CVD	Yes	247	64.8
Do you think that OA is treated with painkillers?	Yes	124	32.5
Joint OA is treated through physical therapy and regular exercise	Yes	300	78.7
OA is treated through interventional treatment with cortisone	Yes	173	45.4
OA is treated through interventional therapy with HA and stem cells	Yes	143	37.5
OA is treated through joint replacement surgery for advanced cases	Yes	266	69.8

As shown in Table 5, the univariate analysis revealed notable differences in awareness scores about OA and its relationship to CVD among participants based on various sociodemographic factors. Notably, age had a significant impact, with younger participants (18-27 years) having the highest mean score (18.27, SD = 6.63) and those over 58 years having the lowest (14.18, SD = 5.89) (p < 0.010). Occupation also significantly influenced awareness, with students scoring the highest (mean = 18.56, SD = 6.28) and the unemployed scoring the lowest (mean = 14.50, SD = 5.34) (p < 0.001). Participants studying or working in healthcare had significantly higher scores (students: mean = 20.46, SD = 5.40; employees: mean = 21.17, SD = 4.94) compared to non-healthcare participants (mean = 15.53, SD = 6.27) (p < 0.001). Receiving information about OA significantly boosted awareness scores (mean = 19.76, SD = 4.776) compared to those who did not receive such information (mean = 14.85, SD = 7.30) (p < 0.001). The source of information was crucial, with school/university sources leading to the highest scores (mean = 21.13, SD = 4.86), and the absence of a source correlating with the lowest scores (mean = 14.42, SD = 7.11) (p < 0.001).

**Table 5:** The difference in awareness scores by various participant features (univariate analysis). ^a^: Mann-Whitney U test; ^b^: Kruskal-Wallis test; SD: standard deviation; N: frequency; Sig: significance; CVD: cardiovascular disease; OA: osteoarthritis

Data		N	Mean	(SD)	Sig. value
Gender	Female	312	17.91	6.39	0.417^a^
	Male	69	17.39	6.29	
Age (years)	18–27	250	18.27	6.63	<0.010^b^
	28–37	44	17.77	6.74	
	38–47	28	17.71	5.90	
	48–57	43	16.62	4.20	
	>58	16	14.18	5.89	
Nationality	Non-Saudi	19	19.10	5.87	0.305^a^
	Saudi	362	17.75	6.39	
Educational status	Secondary/Less	120	17.84	6.42	0.103^b^
	Diploma	33	15.48	6.88	
	Bachelor’s	210	18.06	6.29	
	Postgraduate	18	19.11	5.38	
Occupation	Unemployed	22	14.50	5.34	<0.001^b^
	Housewife	41	16.60	6.71	
	Employee	62	17.80	6.56	
	Student	223	18.56	6.28	
	Retired/Other	31	16.12	5.81	
Study/Work in the health field	No	207	15.53	6.27	<0.001^b^
	Yes (student in healthcare)	157	20.46	5.40	
	Yes (employee in healthcare)	17	21.17	4.94	
Diagnosed with/know someone diagnosed with osteoarthritis	No	229	17.43	6.89	0.369^a^
	Yes	152	18.39	5.45	
If yes, ever been diagnosed with CVD	No	223	17.48	6.62	0.204^a^
	Yes	55	19.05	5.31	
Ever received information related to joint OA?	No	151	14.85	7.30	<0.001^b^
	Yes	230	19.76	4.776	
Source of information	No source	70	14.42	7.11	<0.001^b^
	School/University	92	21.13	4.86	
	Internet/Social media	55	17.50	5.69	
	Family/Friends	34	18.67	5.02	
	Healthcare professionals	28	19.10	4.81	
	Know someone with OA	24	20.66	4.71	
	Personal experience	10	19.90	4.33	

**Figure 2:**
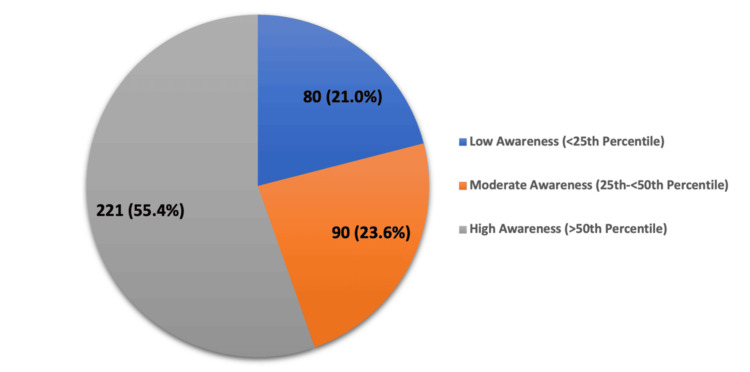
Participants’ awareness of osteoarthritis and its relationship with cardiovascular disease.

Figure 2 shows the distribution of overall awareness about OA and its relationship with CVD among participants. The majority of participants, constituting 211 (55.4%), exhibited high awareness levels, while 90 (23.6%) showed moderate awareness. Only 80 (21.0%) demonstrated low awareness levels, indicating a generally high level of understanding among the participants.

Table 6 presents the results of the multivariate analysis, which identified several significant predictors of high knowledge about OA and its association with CVD. Age, gender, nationality, higher education, and occupation did not show significant predictive value for high knowledge. However, studying or working in healthcare was a significant predictor. Participants studying in the healthcare field had a significantly higher likelihood of possessing high knowledge, with an Exp(B) of 3.325 (p = 0.001), indicating they were over three times more likely to have high knowledge compared to the reference group. While working in the healthcare field also showed an increased likelihood (Exp(B) = 1.941), this was not statistically significant (p = 0.285). Importantly, receiving information related to OA significantly increased the likelihood of high knowledge, with an Exp(B) of 2.222 (p = 0.007), indicating that participants who received information about OA were more than twice as likely to have high knowledge compared to those who did not.

**Table 6:** Adjusted predictors of high knowledge among participants (multivariate analysis). B: regression coefficient; Sig.: significance; Exp(B): exponentiated coefficient; CI: confidence interval; Ref: reference; OA: osteoarthritis; CVD: cardiovascular disease

Predictors	B	Sig.	Exp(B)	95% CI	
				Lower	Upper
Age	-0.115	0.396	0.891	0.682	1.163
Gender (male)	-0.115	0.757	0.892	0.431	1.844
Nationality (Saudi)	-0.363	0.515	0.695	0.233	2.078
Higher education	0.100	0.493	1.105	0.830	1.471
Occupation	0.043	0.750	1.044	0.800	1.364
Study/Work in the health field	Ref	0.004	Ref	Ref	Ref
Study in the health field	1.202	0.001	3.325	1.617	6.838
Work in the health field	0.663	0.285	1.941	0.575	6.556
Diagnosed with/know someone diagnosed with OA	0.169	0.573	1.184	0.658	2.129
If yes, ever been diagnosed with CVD	0.045	0.899	1.046	0.522	2.096
Ever received information related to joint OA?	0.799	0.007	2.222	1.242	3.977
Constant	-1.570	0.091	0.208	-	-

## Discussion

OA is a chronic, degenerative, non-inflammatory, and disabling disease of the joints, characterized by complex disorders of the entire synovial joint [17]. It is a musculoskeletal disorder that often coexists with CVD. Slater et al. (2011) showed that comorbid arthritis and back problems increase activity limitations, with CVD prevalence ratios of 1.60 and 1.46, respectively [18]. Risk factors include age, gender, obesity, and joint injuries. Similarly, Goel et al. (2021) found a strong relationship between knee OA and CVD, with the cardiovascular risk score being positively correlated to the severity of OA [19]. It causes pain and disability among patients, primarily in older age [20]. Our study aimed to investigate the level of awareness regarding lifestyle modifications to control OA and their association with cardiac risk factors by examining sociodemographic factors and information sources. Additionally, the study offers insights for healthcare professionals to enhance patient education and management strategies.

Our study population revealed a predominantly young, female, and Saudi cohort, consistent with previous studies highlighting the higher prevalence of OA among women. Tschon et al. (2021) showed that women use more healthcare services, and have a higher OA prevalence, more pain and inflammation, and greater physical difficulty compared to men [21]. This demographic trend aligns with the global epidemiology of OA, emphasizing the need for targeted interventions and preventive measures, particularly among high-risk populations. Interestingly, age and gender did not significantly predict high knowledge levels in our multivariate analysis. Similarly, a study by Alghamdi et al. (2023) showed no association between knowledge about OA and age or gender [22]. Educational attainment and healthcare-related work or study emerged as strong predictors, underscoring the pivotal role of formal education and healthcare exposure in enhancing awareness of OA and its cardiovascular implications. Alahmed et al. (2023) demonstrated that participants with a university or higher education had a good level of knowledge about OA compared to those with lower levels of education [23]. However, limited knowledge is available on occupation as a predictor of awareness.

Our study revealed diverse channels through which participants obtained knowledge about OA and CVD. School or university education emerged as the most influential source, followed by the internet/social media, family/friends, healthcare professionals, and personal experiences with OA. A previous study by Alahmed et al. (2023) reported good knowledge levels among participants whose information came from healthcare staff compared to those who had no specific source of information (p = 0.003) [23]. Notably, participants who knew someone with OA were over 10 times more likely to have high knowledge levels, emphasizing the significant impact of personal connections and lived experiences in shaping awareness and perceptions of musculoskeletal disorders. Similarly, Alghamdi et al. (2023) found that 435 (40%) participants who knew someone diagnosed with joint roughness had a good awareness level compared to 335 (30.8%) of those who did not (p = 0.001). [22]. These findings highlight the importance of community engagement and peer support networks in disseminating accurate information and promoting proactive management strategies for OA and related comorbidities.

Regarding the assessment of participants’ knowledge of OA causative factors and symptoms, our findings showed varying levels of awareness. Notably, factors such as erosion of cartilage, decreased synovial fluid, and joint injuries were commonly recognized as causes of OA. Silverwood et al. (2014) identified various causative factors for OA, including obesity, female gender, genetics, joint use, bone density, muscle weakness, and laxity, which contribute to OA risk [24]. However, various misconceptions persisted among patients, such as believing that pain is the sole symptom of OA and that joint involvement is universally applicable. Chen et al. (2017) showed that while pain is the main symptom of OA, it is not the only one, as various other symptoms are also present in OA patients [25,26]. These findings highlight the importance of targeted educational initiatives aimed at addressing myths and misconceptions surrounding OA.

Furthermore, our analysis of lifestyle modifications for controlling OA and their relationship to CVD risk factors underscored the intricate link between joint health and cardiovascular wellness. Participants demonstrated awareness of shared risk factors such as obesity, sedentary behavior, and unhealthy dietary habits, emphasizing the need for comprehensive management approaches targeting modifiable risk factors common to both conditions. Notably, healthcare students and professionals exhibited significantly higher knowledge levels, highlighting the pivotal role of formal education and professional training in enhancing awareness and promoting evidence-based practices in musculoskeletal and cardiovascular healthcare settings [27].

Limitations

There are several limitations of our study which include a predominantly young and female sample, potentially limiting generalizability. Self-reported data may introduce bias, and the cross-sectional design prevents causal inference. Additionally, the reliance on specific information sources may not capture the full range of participant knowledge and experiences.

Implications and future direction

Our study emphasizes the importance of targeted education and healthcare exposure in enhancing OA and CVD awareness. Future research should explore broader, more diverse populations and longitudinal designs to establish causal relationships. Integrating patient perspectives into educational strategies can further improve health outcomes and disease management.

## Conclusions

The findings of this study underscore a notable gap in awareness regarding the relationship between OA and CVD among the population in Al Ahsa, Saudi Arabia. While the majority of participants demonstrated a high level of awareness about OA and its associated risk factors, particularly among those with healthcare exposure, there remains a substantial proportion of the population with limited knowledge of the connection between OA and CVD. This highlights the need for targeted educational interventions, particularly in younger and non-healthcare-educated groups, to enhance understanding of the lifestyle modifications that can alleviate both OA and CVD risks. By addressing knowledge gaps and misconceptions, promoting healthy lifestyle behaviors, and fostering collaborative partnerships between patients and healthcare providers, we aim to improve outcomes and enhance the quality of life for individuals living with OA and its associated comorbidities.

## Appendices

Questionnaire

Table 7 shows the first section of the questionnaire, which includes the consent of participation, and the sociodemographic data.

**Table 7:** Section 1: Sociodemographic data and consent.

Sociodemographic data and consent	Choices
Do you agree to participate in this survey?	Yes, No
Age	18–27, 28–37, 38–47, 48–57, 58 or older
Gender	Female, Male
Nationality	Saudi, Non-Saudi
Educational level	I did not receive any education, Elementary, Middle school, High school, Diploma, Bachelor, Master, PhD, Other
Employment status	Employed, Non-Employed, Student, Housewife, Retired, Craftsman/Manual worker, Other
Are you studying or working in the health field?	Yes a student in the health field, Yes a worker in the health field, No, Other
Have you been diagnosed/know someone who has been diagnosed with osteoarthritis?	Yes, No
Have you ever heard or read anything about osteoarthritis?	Yes, No
If the answer was yes, what sources did you get the information from?	Personal experience, Friends and family, Internet and social media, Health service providers, School/University, Media, I know a person with osteoarthritis, I don’t have a source

Table 8 shows the second section of the questionnaire, which measures the participants’ knowledge regarding factors leading to the development of osteoarthritis.

**Table 8:** Section 2: Factors leading to the development of osteoarthritis.

Questions	Choices
Do you think erosion of the cartilage surrounding the joint is among the causes of osteoarthritis?	Yes, No, I don’t know
Do you think decreased blood around the joint is among the causes of osteoarthritis?	Yes, No, I don’t know
Do you think a decreased amount of synovial fluid in the knee with increased movement is among the causes of osteoarthritis?	Yes, No, I don’t know
Do you think increased use of joints among manual workers is among the causes of osteoarthritis?	Yes, No, I don’t know

Table 9 shows the third section of the questionnaire, which measures the participants’ knowledge of the symptoms of osteoarthritis.

**Table 9:** Section 3: Symptoms of osteoarthritis.

Questions	Choices
Do you think that pain is the only symptom of osteoarthritis?	Yes, No, I don’t know
Do you think swelling is a sign of osteoarthritis?	Yes, No, I don’t know
Do you think that stiffness is a symptom of osteoarthritis?	Yes, No, I don’t know
Do you think osteoarthritis can lead to loss of joint movement?	Yes, No, I don’t know
Do you think that all joints in the body can get osteoarthritis?	Yes, No, I don’t know

Table 10 shows the fourth section of the questionnaire, which measures the extent of participants’ knowledge of the risk factors for developing osteoarthritis and their relationship to cardiovascular diseases.

**Table 10:** Section 4: Risk factors of osteoarthritis and their relationship to cardiovascular diseases.

Questions	Choices
Do you think osteoarthritis is related to age?	Yes, No, I don’t know
Do you think osteoarthritis is more associated with women?	Yes, No, I don’t know
Do you think genetic factors can be a risk factor for developing osteoarthritis?	Yes, No, I don’t know
Do you think previous joint injuries could be a risk factor for developing osteoarthritis?	Yes, No, I don’t know
Do you think joint surgeries can be a risk factor for developing osteoarthritis?	Yes, No, I don’t know
Do you think there is a relationship in terms of risk factors between heart disease and osteoarthritis?	Yes, No, I don’t know
Do you think muscle weakness and joint stiffness can be a reason for lack of motor activity and thus lead to cardiovascular diseases?	Yes, No, I don’t know
Do you think obesity can lead to osteoarthritis of the joints and thus lead to cardiovascular diseases?	Yes, No, I don’t know
Do you think that following an unhealthy diet and not drinking water can be a cause of joint stiffness and thus lead to cardiovascular disease?	Yes, No, I don’t know
Do you think that high levels of cholesterol in the blood can be a cause of cardiovascular disease and osteoarthritis as well?	Yes, No, I don’t know
Do you think that high blood sugar levels could be a risk factor for heart disease and osteoarthritis as well?	Yes, No, I don’t know
Do you think that smoking or sitting with smokers can be a cause of cardiovascular disease and osteoarthritis as well?	Yes, No, I don’t know

Table 11 shows the final section of the questionnaire, which measures the participants’ behavior and response to the symptoms of osteoarthritis.

**Table 11:** Section 5: Behavior and response to symptoms of osteoarthritis.

Questions	Choices
Do you think osteoarthritis is diagnosed through clinical examination and regular diagnostic X-rays?	Yes, No, I don’t know
Do you think osteoarthritis can be diagnosed using laboratory tests?	Yes, No, I don’t know
Do you think that treating osteoarthritis is achieved by reducing excess weight, following a healthy diet, and drinking plenty of water?	Yes, No, I don’t know
Do you think that controlling the risk factors that cause joint osteoarthritis reduces the incidence of heart disease?	Yes, No, I don’t know
Do you think that osteoarthritis is treated with painkillers?	Yes, No, I don’t know
Do you believe that joint osteoarthritis is treated through physical therapy and regular exercise?	Yes, No, I don’t know
Do you think that osteoarthritis is treated through interventional treatment with cortisone?	Yes, No, I don’t know
Do you think that osteoarthritis is treated through interventional therapy with hyaluronic acid and stem cells?	Yes, No, I don’t know
Do you believe that osteoarthritis is treated through joint replacement surgery in advanced cases?	Yes, No, I don’t know
